# Overexpressed DNA Polymerase Iota Regulated by JNK/c-Jun Contributes to Hypermutagenesis in Bladder Cancer

**DOI:** 10.1371/journal.pone.0069317

**Published:** 2013-07-26

**Authors:** Fang Yuan, Zhigang Xu, Mingzhen Yang, Quanfang Wei, Yi Zhang, Jin Yu, Yi Zhi, Yang Liu, Zhiwen Chen, Jin Yang

**Affiliations:** 1 Urology Institute of People Liberation Army, Southwest Hospital, The Third Military Medical University, Chongqing, China; 2 Department of Clinical Biochemistry, The Third Military Medical University, Chongqing, China; 3 Department of Cell Biology, The Third Military Medical University, Chongqing, China; University of Manitoba, Canada

## Abstract

Human DNA polymerase iota (pol ι) possesses high error-prone DNA replication features and performs translesion DNA synthesis. It may be specialized and strictly regulated in normal mammalian cells. Dysregulation of pol ι may contribute to the acquisition of a mutator phenotype. However, there are few reports describing the transcription regulatory mechanism of pol ι, and there is controversy regarding its role in carcinogenesis. In this study, we performed the deletion and point-mutation experiment, EMSA, ChIP, RNA interference and western blot assay to prove that c-Jun activated by c-Jun N-terminal kinase (JNK) regulates the transcription of pol ι in normal and cancer cells. Xeroderma pigmentosum group C protein (XPC) and ataxia-telangiectasia mutated related protein (ATR) promote early JNK activation in response to DNA damage and consequently enhance the expression of pol ι, indicating that the novel role of JNK signal pathway is involved in DNA damage response. Furthermore, associated with elevated c-Jun activity, the overexpression of pol ι is positively correlated with the clinical tumor grade in 97 bladder cancer samples and may contribute to the hypermutagenesis. The overexpressed pol ι-involved mutagenesis is dependent on JNK/c-Jun pathway in bladder cancer cells identifying by the special mutation spectra. Our results support the conclusion that dysregulation of pol ι by JNK/c-Jun is involved in carcinogenesis and offer a novel understanding of the role of pol ι or c-Jun in mutagenesis.

## Introduction

Cells utilize DNA repair pathways to efficiently correct the deleterious effects of DNA damage. However, in some cases, not all damage can be repaired promptly and availably, which induces cell cycle arrest. To overcome this blockade to continue synthesis of the growing DNA chain, cells employ the molecular mechanisms that enable translesion DNA synthesis (TLS) to occur [1], [2]. TLS is performed by specialized DNA polymerases, including Y-family DNA polymerases (pol ι, pol κ, pol η and Rev1), which commonly exhibit high error rates during DNA synthesis. It has been demonstrated that pol ι has the lowest fidelity in TLS process across many types of DNA lesions *in vitro* and pol ι may also replicate undamaged DNA with low fidelity [3].

As far as the error-prone DNA replication features of pol ι, dysregulation of pol ι may contribute to the acquisition of mutator phenotype that, along with defective cell cycle control or others genome stability pathways, could facilitate to accelerate the tumor progression. We previously first discovered that breast cancer cells overexpress pol ι protein, which leads to UV-induced hypermutagenesis [4]. pol ι is also involved in UV-induced mutagenesis in Burkitt's lymphoma and XPV cell lines [5], [6]. Moreover, pol ι was preliminary found to be overexpressed in a variety of primary tumor tissues, including prostate, uterus, stomach and rectal cancers, but there is a lack of the valuable clinical evidence [7]. However, the role of pol ι in carcinogenesis is still obscure and under debating [8] and few reports are concerned about the regulatory mechanism of pol ι or the potential dysregulation mechanism of pol ι in cancer. Therefore, it is important to make our efforts to clarify the mechanism of regulation and to determine the carcinogenesis role of pol ι *in vivo*.

A prominent signaling pathway activated in response to cellular stress is the mitogen-activated protein kinase (MAPK) pathway. Three major MAPK pathways have been described: the extracellular signal-regulated kinase (ERK), c-Jun N-terminal kinase (JNK) and p38 MAPK pathways. Targets of JNK pathway include the activator protein 1 (AP-1) group of transcription factors, such as the Jun, Fos and ATF family members [9]. Nevertheless, c-Jun specifically phosphorylated by JNK plays a central role in diverse functions of AP-1 complex [10]. JNK can be activated by cellular stress signals resulting from inflammatory cytokines and DNA damage agents, whereas AP-1 can impact the cellular DNA damage response by regulating the expression of several genes relevant to the DNA damage response mechanisms [11], [12], [13]. Former evidence has also shown that c-Jun contributes to transformation and tumor development and is classified as a proto-oncogene [14]. JNK activation has been demonstrated as a requirement for the development of various cancers [15], [16], [17]. However, no study has yet been reported that elucidates the role of JNK/c-Jun pathway in TLS and DNA damage-induced hypermutagenesis. Moreover, most reports describing the signaling for JNK pathway activation following DNA damage have focused on membrane receptor-related JNK activation. The manner by which DNA damage response-related mechanisms contributes to JNK activation in nucleus is currently unclear.

Our studies provide the evidence that JNK/c-Jun pathway plays a crucial transcription regulatory role of pol ι. The data also furnish a novel insight that JNK/c-Jun pathway is involved in TLS and hypermutagenesis. We determined that JNK/c-Jun pathway can be activated by DNA damage key proteins, XPC and ATR. In addition, overexpression of pol ι was found to be related to the extent of c-Jun phosphorylation in bladder cancer tissues and be associated with cancer progression and hypermutagenesis identified by the special mutation spectra. It is suggested that dysregulated pol ι by JNK/c-Jun may function as a mutator. Therefore, DNA polymerase iota may be a potential indicator and target for anticancer treatment in malignant tumor.

## Materials and Methods

### Ethics Statement

The research was approved by the ethics board of Southwest Hospital at the Third Military Medical University (KY201016), and all participants provided written informed consent.

### Cell culture and treatment

T24, RT4 bladder carcinoma cells and HEK293 cells were purchased from American Type Culture Collection (ATCC, Rockville, MD, USA). Cells were maintained in RPMI 1640, McCoy's 5A or DMEM medium supplemented with 10% FBS and a mixture of antibiotics, respectively. Inhibitors of JNK (SP600125), p38 (SB203580) and ERK1/2 (PD98059) (Sigma-Aldrich, St Louis, MO, USA) were used for treating the cells for 2 hours. Exposure of cells and plasmids to UV light (254 nm) was achieved with a CL-1000 ultraviolet crosslinker.

### Clinical samples and immunohistochemistry

Urothelial carcinoma tissue samples were obtained from 97 patients by the transurethral bladder tumor resection and radical cystectomy at the Southwest Hospital. All the samples were collected between 2008 and 2010. These tissues were assigned pathological grades of urothelial carcinoma I, II or III according to the WHO classification (1973). The formalin-fixed, paraffin-embedded tissue sections were deparaffinized, rehydrated in graded alcohols series, and processed using the streptavidin immunoperoxidase method. An anti-pol ι antibody (LS-B296; Lifespan Biosciences, Seattle, WA, USA) and an anti-p-c-Jun antibody (sc-822; Santa Cruz Biotechnology, CA, USA) were used at dilutions of 1∶200 and 1∶100, respectively. The protein expression levels were quantified and scored by evaluating the antibody signal strength and estimating the percentage of positively stained urothelial carcinoma cells [18]. Five random fields of view were chosen, and 100 cells per field were analyzed. Scoring was performed blind without clinical data. The scoring was cataloged as 1 score (percentage of positively stained cells<25%), 2 score (25–50%), 3 score (50–75%) and 4 score (>75%), respectively. Meanwhile, the stain signal was graded as weak (1 score), moderate (2 score) and strong (3 score), respectively. The percentage score multiplying the signal score was final score. The final score was defined low level (<6 score) and intense level (>8 score).

### Western blot assay

Equal proportions of each cells lysate were analyzed by SDS-PAGE [19]. The antibodies were contained anti-pol ι (ab1324; Abcam, Inc., Cambridge, UK), anti-XPC (ab6264; Abcam), anti-ATR (sc-1887; Santa Cruz), anti-c-Jun (sc-44X; Santa Cruz), anti-p-c-Jun (sc-822; Santa Cruz), anti-p-MKK4 (BS4168; Bioworld, St. Louis Park, MN, USA), anti-p-MKK7 (4171; Cell Signaling Technology, Boston, MA, USA), anti-p-JNK (sc-6254; Santa Cruz), anti-JNK (9252; Cell Signaling Technology), anti-MKP-1(sc-1102; Santa Cruz), and anti-GAPDH (KC-5G4; Kangchen, Shanghai, China). Protein expression was evaluated using the SuperSignal West Pico kit (NCI4106; Pierce, Rockford, IL, USA). The experiment was repeated at least three times. The level of protein expression was quantified using Scion image densitometry software (Scion Corporation, Frederick, MD).

### Construction of pGL3–*POLI* deletion and site-directed mutagenesis

The −2751/+63, −1832/+63, −1299/+63, −685/+63, −275/+63, −208/+63, −160/+63 and −126/+63 of DNA fragments of human *POLI* gene (relative to the transcriptional start site) were amplified from HEK293 genomic DNA via PCR. Mutagenic primers were designed for the c-Jun binding site within −275/+63 with a 2 bp mismatch (bolded and underlined); forward: 5′-TGAGCCGGTATTGC**CA**CACTGTTGCCCAC-3′ and reverse: 5′-CTGCAACCTCTGCCTCCCGGATTCAAGC-3′. The amplified PCR products were then inserted into the *KpnI* and *HindIII* sites of pGL3-basic vector (Promega, Madison, WI, USA). All the constructs were confirmed by DNA sequencing (Invitrogen, Shanghai, China).

### Transient transfection and luciferase assays

The cells (1×10^5^) were either transfected with different subcloned luciferase reporter plasmids and pSV-β-Galactosidase or collectively transfected with pcDNA3.1-c-Jun using the Lipofectamine transfection reagent (Invitrogen). The pGL3-basic and empty pcDNA3.1 vectors were used as controls. At 48 hours post-transfection, the cells were harvested and analyzed using Luciferase Assay System (E1500; Promega). The pSV-β-gal (E2000; Promega) was analyzed as a control. The assay was performed in triplicate, and each experiment was repeated at least three times.

### Electrophoretic mobility shift assays

The experiments were performed as described earlier, but with some modifications [20]. The oligonucleotides were labeled with a Biotin 3′ End DNA Labeling Kit (89818; Pierce). The biotin-labeled 29 bp oligos (5′-TGAGCCGGTATTGCGTCACTGTTGCCCAC-biotin-3′; AP-1-like binding site indicated in underlined) and the mutant double-stranded 29 bp oligos (5′-TGAGCCGGTATTGC**CA**CACTGTTGCCCAC-3′; mutation site bolded and underlined) were used to test for specific binding. Non-labeled oligos were used for competition. The LightShift Chemiluminescent EMSA Kit (20148; Pierce) was used to perform the reactions. The labeled oligos (20 fmol) were incubated with nuclear extracts (4 µg) from HEK293 cells. Following electrophoresis in non-denaturing 6% polyacrylamide gel, the gel was dried and subjected to chemiluminescence. Supershift assays were performed using an anti-c-Jun antibody (sc-44X; Santa Cruz) and a mouse IgG (sc-2025; Santa Cruz).

### Chromatin immunoprecipitation assay

ChIP was performed as previously described [21]. Briefly, after cross-linking with 1% formaldehyde, HEK293 cells were lysed and sonicated. Prior to immunoprecipitation with mouse IgG or c-Jun (sc-44X, Santa Cruz) antibodies, a small aliquot of chromatin was saved and used as an input control. The specific targeted DNA fragment was measured with specific primers (forward: 5′-GCCGGTATTGCGTCACTGTT-3′; reverse: 5′-CAGAGTGACACGCATGCCAAGGAATCG-3′) to amplify the −236/−61 region of *POLI* gene.

### Bioinformatics analysis

Approximately 3,000 bp of the 5′ flanking region of the *POLI* sequence was obtained using the BLAST algorithm of the National Center for Biotechnology Information (http://www.ncbi.nlm.nih.gov/ and http://genome.ucsc.edu/cgi-bin/hgc). Putative transcription factor binding sites were identified with TransFac professional 8.1 software (http://www.cbrc.jp/research/db/TFSEARCH.html). A promoter prediction analysis was performed using an online database (http://fruitfly.org/seq_tools/ promoter.html).

### RNA interference experiments

c-Jun siRNA (sc-29223; Santa Cruz) which is a pool of 4 target-special 19–25 nt siRNAs, ATR siRNA (sc-29763; Santa Cruz) which is a pool of 3 target-special 19–25 nt siRNAs or control siRNA-A (sc-37007; Santa Cruz) diluted in transfection medium (sc-36868; Santa Cruz) was transfected into cells by transfection reagent (sc-29528; Santa Cruz). XPC-specific shRNA (5′-GGATGAAGCCCTCAGCGAT-3′) [22] and non-specific control shRNA were synthesized and inserted into a lentivirus vector (Genechem, Shanghai, China), then infected cells, respectively. The experiments were independently preformed at least three times.

### Detection of UVC-mediated mutations in *SupF* reporter gene

The plasmid pSupFG1 has been described previously [23], [24]. Briefly, the UV-damaged plasmid (10 µg) was transfected into cells pre-treated with or without SP600125. Plasmid DNA was isolated after 48 hours and transformed into an *E. coli* SY204 (lacZ amber) strain via electroporation at 1800 V/20 mF. The mutation frequency in the *SupF* reporter gene was determined as the number of mutant colonies (white) on agar plates dividing the number of total colonies. The mutation spectrum of *SupF* gene in white colonies was demonstrated by DNA sequencing. The experiment was repeated at least four times.

### Real-time PCR

Total RNA was isolated using TRIzol and reverse-transcribed to generate cDNA (Invitrogen). Amplification curves were generated by monitoring the fluorescence of SYBR Green (Invitrogen). The primers used for amplification included the following: MKP-1 forward, 5′-GTACATCAAGTCCATCTGAC-3′ and reverse, 5′-GGTTCTTCTAGGAGTAGACA-3′; GAPDH forward, 5′-AAGGTCGGAGTCAACGGATT-3′ and reverse, 5′-CTCCTGGAAGATGGTGATGG-3′. The assay was performed in triplicate, and each experiment was repeated at least three times.

### Statistical analysis

Data were collected at least in triplicate and expressed as mean ± standard error (SE). Differences between groups for densitometry of molecular expression were analyzed by the Student's t-test. The statistical significance between pol ι expression and pathological grade of bladder tumor tissues was estimated using a chi-square test. The correlation between pol ι and p-c-Jun expression was analyzed with a bivariate test. SPSS13.0 software was used to conduct the statistical analysis (p<0.05 was considered significant). The Immunoblot, EMSA and ChIP analysis data were representative of at least three to five independent studies with reproducible results.

## Results

### c-Jun is a critical transcriptional factor for *POLI* gene

**Figure 1:**
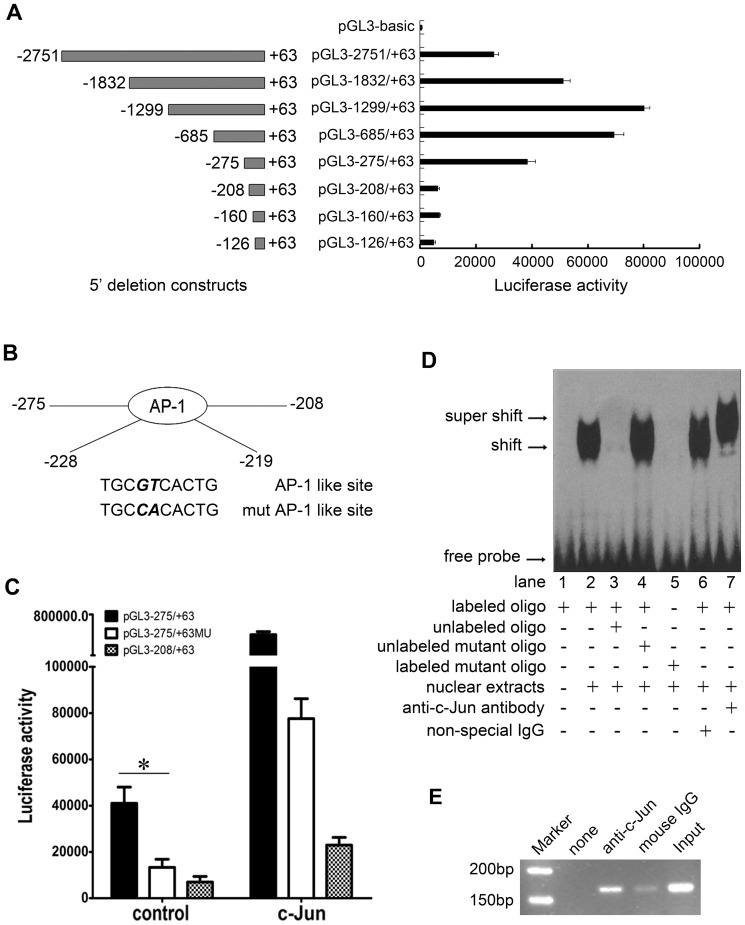
c-Jun activates and binds to the human *POLI* promoter. (A) Luciferase activity of the deletions of 5′ flanking region of *POLI* gene was normalized by β-*gal* activity. Each bar represents the mean±SD for at least three independent experiments. (B) Schematic representations of the distal *POLI* promoter region. (C) Transcriptional activities of the deletion or mutant of *POLI* promoter. *Statistically significant difference compared to the pGL3-275/+63 and pGL3–275/+63MU construct (p<0.01; Student's t-test). (D) EMSA Assay. (E) ChIP assay. Mouse IgG as a negative control. The Input DNA or no DNA added was each used as positive and blank control, respectively.

Considering the experimental clues showed that altered expression of pol ι may be associated to hypermutagenesis and carcinogenesis, we firstly clarify its transcriptional regulation mechanism. To map the minimal promoter activity and the regulatory sequences, we analyzed human *POLI* gene by bioinformatics and identified the basic promoter of *POLI* gene with a putative AP-1 *cis*-element. A series of deletions at the 5′flanking region of *POLI* gene were subcloned into pGL3-basic vector (a luciferase reporter vector). The primers were listed in Table 1. The activities of these constructs were assessed based on the luciferase activity following transient transfection into HEK293 cells, a commonly used cells line as tool cells. Our results showed that the minimal *POLI* promoter is located at the −275/+63 region (Fig. 1A). Further analyses demonstrated that the region (−275/−208) contains a putative AP-1-like binding *cis*-element (TGCGTCA) at the −228 site, which is similar with the highly evolutionarily conserved AP-1 binding sequence of TGAGTCA (Fig. 1B).

**Table 1:** Primers used in PCR for *POLI* gene.

Name	Sequences	Site
F1	5′ CACTTgCTAAATACTTTCTggggTCCAgg 3′	−2751
F2	5′ AgACTTTgAAAgAACTggTCCAgACATC 3′	−1832
F3	5′ TAACgCACTTggATCATCCCAAAACCAT 3′	−1299
F4	5′ CAACAAACAAAACACAgCAAACCTTAAC 3′	−685
F5	5′ ggAgAATCgCTTgAATCCgggAggCAg 3′	−275
F6	5′ CAgCCTgAgCAACAAgAACgAAACT 3′	−208
F7	5′ gCCATTTgTgCTTTCAATCTCTCCgCT 3′	−160
F8	5′ TCCACCCggCgggAAAAAAC 3′	−126
R	5′ CgCTgCCAACCgCAgCgCTACTTCC 3′	+63

Furthermore, to clarify the regulatory transcriptional role of this binding *cis*-element, we performed a mutagenesis analysis of the −275/+63 region of *POLI* gene. A vector designated pGL3-275/+63MU (a mutated AP-1-like binding motif) was constructed with two point mutations (TGCcaCA). Intriguingly, luciferase activity was severely attenuated in pGL3-275/+63MU (Fig. 1C, left column). c-Jun plays a central role in diverse functions of the AP-1 complex, therefore, we transiently co-transfected HEK293 cells with the constructs and pcDNA3.1-c-Jun to determine whether c-Jun could activate the *POLI* promoter. The result of co-transfection revealed that luciferase was the most significantly activated in pGL3-275/+63, although pGL3-275/+63MU and pGL3-208/+63 was observed to be also increased (Fig. 1C, right column). It is possible that the sequence may contain additional regulatory elements indirectly controlled by the overexpressed c-Jun that were not identified by our study. Nevertheless, our data still suggest that c-Jun can efficiently activate the transcription of *POLI* gene *via* the AP-1 *cis*-element at the −228 site.

To verify that c-Jun directly binds to the motif in the *POLI* promoter, we performed EMSA using nuclear extracts from HEK293 cells. A shifted band was specially produced and supershift analysis with an anti-c-Jun antibody demonstrated that the band actually contained c-Jun protein (Fig. 1D). To obtain evidence of c-Jun directly binding to the region *in vivo*, ChIP was also performed with an anti-c-Jun antibody to precipitate chromatin from HEK293 cells (Fig. 1E). In combination with the above findings, c-Jun appears to directly bind to the *POLI* promoter and constitutively activate the expression of pol ι in HEK293 cells.

### Overexpression of pol ι is depended on activated JNK/c-Jun

It is well documented that translesion DNA polymerases, including pol ι, may play an important role in DNA damage response. Up to date, the certain types of DNA damage which is able to be bypassed by pol ι are still obscure and controversial. We examined DNA damage-induced expression of pol ι, which was not observed to be induced by some types of DNA damage, including cisplatin, bleomycin and etoposide (unpublished data). However,many evidences support the notion that one of the predominant characteristics of pol ι is the propensity to incorrectly incorporate the nucleotides opposite some types of DNA lesions, which include cyclobutane pyrimidine dimers and 6-4 pyrimidine pyrimidone photo adduct distortion following UV damage [8], [25]. More importantly, we proved that pol ι can be significantly induced upon UV damage in previous study [4].

**Figure 2:**
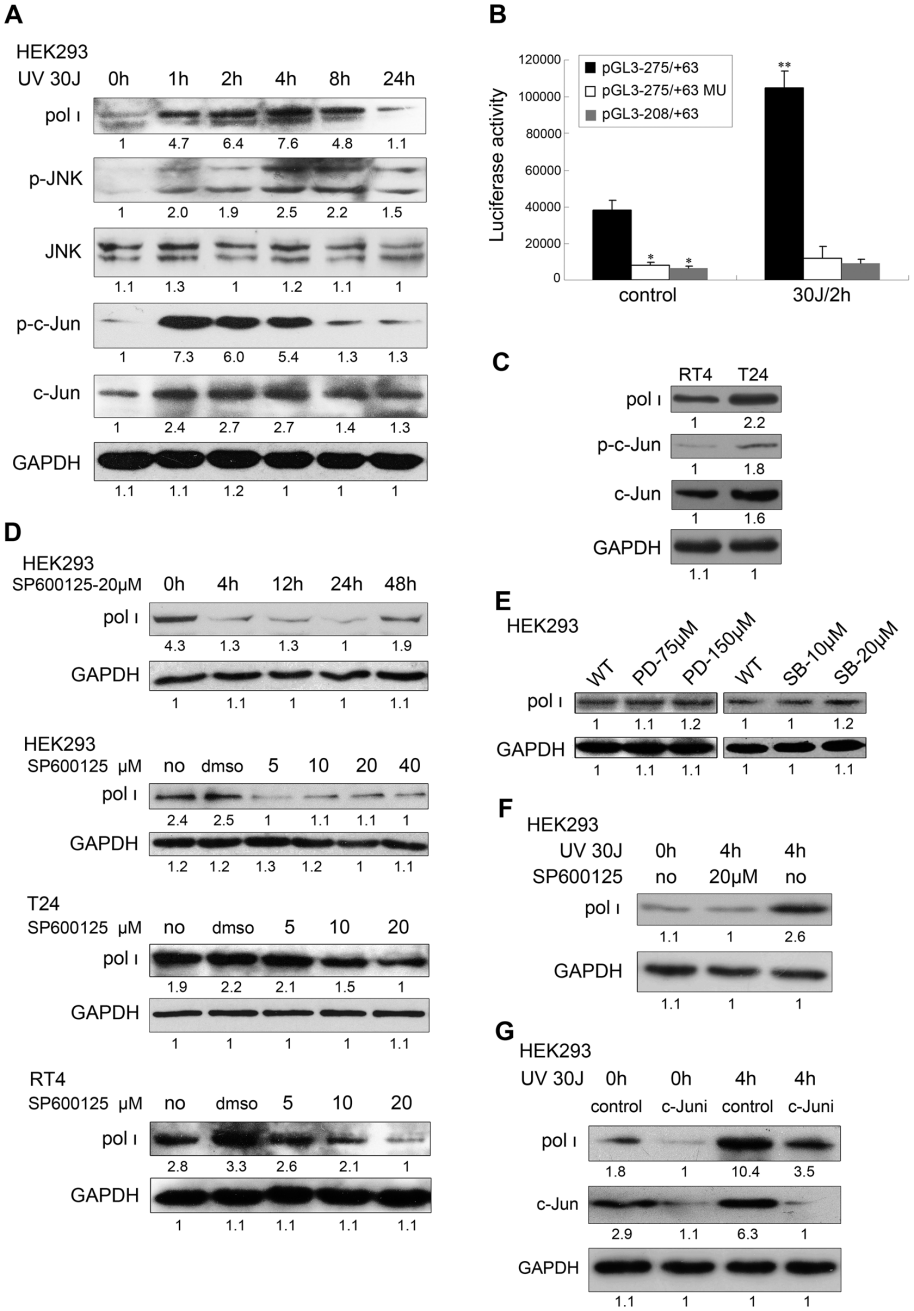
The expression of pol ι is dependent on activated JNK/c-Jun. (A) The kinetics of pol ι, p-JNK and p-c-Jun expression under UV damage in HEK293 cells. (B) The effect of UV damage on the activity of the *POLI* promoter region. *Statistically significant difference compared to the pGL3-275/+63 and pGL3–275/+63MU construct (p<0.01; Student's t-test). **Statistically significant difference compared to the cells treated with or without UV (p<0.01). (C) The expression of pol ι, c-Jun and p-c-Jun in RT4 and T24 bladder cancer cells. The expression of pol ι was observed in (D) HEK293, T24 and RT4 cells treated with different concentrations of SP600125 cultured for additional different lengths of time; (E) HEK293 cells treated with PD98059 or SB203580; (F) HEK293 cells treated with or without SP600125 and UV; (G) HEK293 cells transfected with siRNA-c-Jun for 48 hours. The non-specific siRNA was used for control. Densitometric values were indicated below each panel.

In order to define the role of JNK/c-Jun in the pol ι-mediated TLS, we chose UV irradiation (UVC 254 nm) as the study mode of DNA damage. To determine if UV-induced pol ι expression is controlled by JNK/c-Jun activity, we verified that the expression of pol ι was rapidly induced by the exposure of HEK293 cells to UVC light of 30 J/m^2^ (a middle appropriate dose [26]) and remained elevated for 2–8 hours, which was concomitant with the activation of c-Jun and JNK following UV irradiation (Fig. 2A). The promoter activity of the pGL3-275/+63 construct was also increased approximately 3 fold after 2 hours of UV damage at the same dose (Fig. 2B).

In light of the findings, we sought to confirm whether JNK/c-Jun is the key regulation factor of pol ι expression in general mechanism. We compared the different expression of pol ι, c-Jun and p-c-Jun in RT4 (a well-differentiated cell line from low-grade tumor) and T24 (a cell line represented a high-grade invasive tumor) bladder cancer cells. As shown in Fig. 2C, the expression of pol ι in T24 cells was notable higher than that in RT4, whereas the expression of c-Jun and the activity of p-c-Jun were enhanced as well. The time- or dose-dependent inhibitions of pol ι were observed in HEK293, T24 and RT4 bladder cancer cells pre-treated for 2 hours with a JNK-specific inhibitor (SP600125) (Fig. 2D), even though the expression of pol ι in HEK293 cells appeared to increase at 48 hours. It may be the inhibition of SP600125 to the JNK activity lasting only for 48 hours, which cause the expression of pol ι recovered at 48 hour after SP600125 treatment. As a control, the basal pol ι expression was not observed alteration in HEK293 cells pre-treated with PD98059 and SB203580 (specific inhibitor of ERK and p38, respectively) (Fig. 2E). The results show that activation of JNK/c-Jun pathway is not only responsible for pol ι expression in normal cells, but also contributes to the potential dysregulation of pol ι in bladder cancer cells. We further demonstrated that JNK/c-Jun also directly regulate the basal and UV-induced expressions of pol ι. When HEK293 cells were pre-treated with SP600125 (20 µM), the expression of pol ι was reduced at 4 hours following UV exposure of 30 J/m^2^ (Fig. 2F). And c-Jun knockdown markedly reduced the basal and UV-induced expression of pol ι (Fig. 2G). Collectively, these data suggest that the overexpression of pol ι is largely depended on the activated JNK/c-Jun pathway.

### ATR and XPC promote p-JNK to regulate pol ι expression in response to UV damage

**Figure 3:**
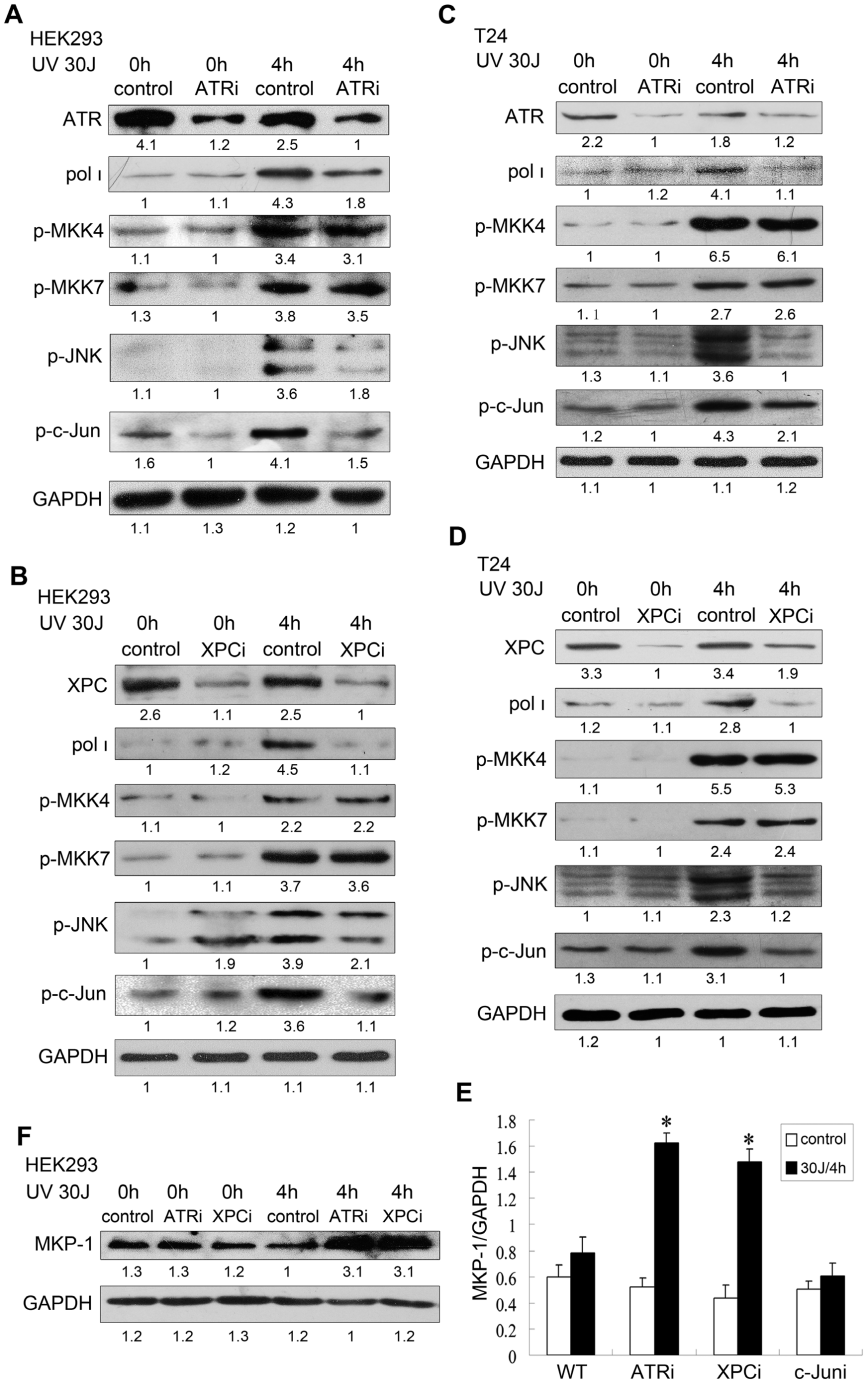
Inhibition of ATR or XPC reduced UV-induced pol ι expression and abolished JNK/c-Jun activation. Protein expression was investigated in different cells with different treatment. (A) HEK293 cells transfected with siRNA-ATR. (B) HEK293 cells infected with shRNA-XPC. (C) T24 cells transfected with siRNA-ATR. (D) T24 cells infected with shRNA-XPC. (E) Real-time PCR analysis of the expression of MKP-1 in untreated or treated HEK293 cells with siRNA-c-Jun, siRNA-ATR or shRNA-XPC; GAPDH mRNA levels were determined as an internal control. *Statistically significant difference compared to HEK293 cells with or without treatment (p<0.01; Student's t-test). (F) MKP-1 protein expression in HEK293 cells treated with siRNA-ATR or shRNA-XPC. The non-specific siRNA or non-specific shRNA-lentivirus was used for control. Densitometric values were indicated below each panel.

Our finding above prompted us to examine the upstream MAPK signaling. A role of DNA damage response signaling has emerged, which can, in addition to well-known indirect membrane or cytoplasmic signaling, activate the MAPK pathway through nuclear signaling [27]. Therefore, we developed the hypothesis that JNK/c-Jun pathway responsible for UV-induced pol ι expression may also be regulated by the DNA damage response. We evaluated the effect of DNA damage response proteins XPC and ATR on the extent of JNK phosphorylation and pol ι expression with UV damage. With siRNA-ATR or shRNA-XPC, the silenced HEK293 and T24 cells both expressed lower levels of pol ι following UV damage, and p-JNK was correspondingly reduced. On the contrary, in the absence of UV damage, the expression of pol ι or p-JNK was not significantly altered by ATR and XPC inhibition, although the p-JNK was observed to be slightly increased by shRNA lentivirus system (Fig. 3A, 3B, 3C and 3D). This result may be explained by the potential specific cellular stress reaction of the lentivirus system. Thus, ATR and XPC are involved in UV-induced JNK signaling, which regulate the expression of pol ι in normal cells and bladder cancer cells.

In the MAPK regulatory network, JNK is phosphorylated and activated by the dual-specificity MAPKKs, including MKK4/SEK1 and MKK7. Sustained JNK activity has been attributed to the impaired dephosphorylation of JNK by MAP kinase phosphatase-1 (MKP-1) [28]. To assess how ATR and XPC activate the JNK pathway, we investigated whether MAPKK signaling regulated by ATR and XPC in response to UV damage. We first determined the extent of UV-induced phosphorylation of MKK4 and MKK7. Relative to wild-type cells, no clear change was observed about the expression of p-MKK4 or p-MKK7 in ATR- and XPC-silenced HEK293 and T24 cells; this may reduce the possible involvement of ATR and XPC in directly activating MAPKK signaling (Fig. 3A, 3B, 3C and 3D), whereas the expression of MKP-1 was significantly increased in the ATR- and XPC-silenced HEK293 cells following UV damage (Fig. 3E and 3F). Collectively others results, our findings imply that DNA damage response protein ATR and XPC influence JNK pathway and regulate the transcription of pol ι by reducing the inhibitory signal MKP-1 rather than activating MKK4 and MKK7.

### Elevated expression of pol ι is associated with activity of c-Jun and malignancy in human bladder cancer

**Figure 4:**
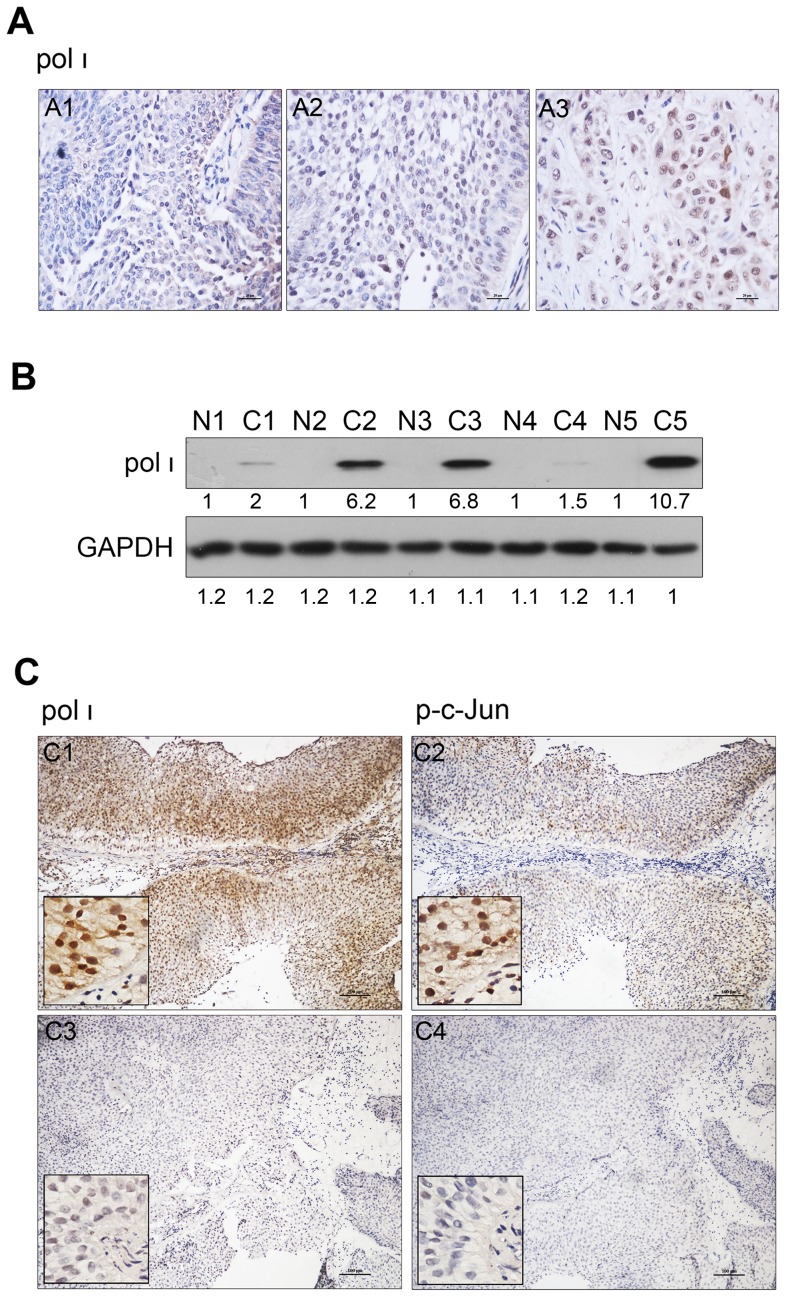
The expressions of pol ι and p-c-Jun in human urothelial carcinoma samples. (A) pol ι protein is aberrantly expressed in different tumor grade according to the WHO classification. A1 is I-grade tumor; A2 is II-grade tumor; A3 is III-grade tumor (×400 magnification). (B) The expression of pol ι in 5 pairs of bladder carcinoma sides and cancer tissues. “N” was represented carcinoma sides tissue, “C” was represented cancer tissue. Densitometric values were indicated below each panel. (C) Representative cases of the concordance of pol ι and p-c-Jun expression in bladder cancer. C1 and C2 were respectively showed the high expression of pol ι and p-c-Jun in a pairs of simple. C3 and C4 were the low expression in another pairs (×100 and ×400 magnification).

Urothelial cells are continuously exposed to many types of DNA damage reagents which are present in urine from industrial aromatic amines, cigarette smoke and the uptake of chemical drugs [29]. Dysfunction of DNA damage response has been implicated as a crucial factor underlying the emergence of genetic instability in urothelial carcinogenesis. Moreover, pol ι which is the propensity to incorrectly incorporate the nucleotides opposite of DNA lesions is abnormal expression in many cancers, although the mechanisms remain unclear. Accordingly, we made an effort to determine whether pol ι plays the potential mutagenic role in bladder cancer which is high heterogeneity and recurrence [29]. We examined the pol ι expression in 97 bladder tumor tissues by immunohistochemical analysis. pol ι protein was mainly localized in nuclei, and a small amount was observed in the cytoplasm. This result revealed that pol ι expression was significantly higher in high-grade bladder tumors than in low-grade tumors (Fig. 4A). Statistical analysis indicated that a strong association existed between pol ι expression and the pathological grade of bladder tumor tissues (Table 2; p<0.01). pol ι expression was also observed to be elevated in bladder cancer tissues compared to carcinoma sides tissues by western blotting (Fig. 4B). These results suggest that the expression of pol ι may serve as an indicator of the malignancy and progression of urothelial carcinomas.

**Table 2:** The expression of pol ι is associated with malignancy in human bladder cancers.

Grade	Low (L; %)	Intense (I; %)	Total No.
I	19 (65.5)	10 (34.5)	29
II	24 (52.2)	22 (47.8)	46
III	3 (13.6)	19 (86.4)	22
No.	46	51	97

pol ι expression were examined in 97 bladder tumor tissues. “Low (L; %)” or “Intense (I; %)” was respectively represented the number and the percentage in the grade of low or high expression of pol ι. The number of samples and the percentage were listed. Statistically significant difference was analyzed by chi-square test to compare to the different pathological grade of bladder tumor tissues (p<0.01).

To determine whether overexpression of pol ι results from dysregulation of JNK/c-Jun activation *in vivo*, we analyzed the concordance of elevated pol ι and abnormal c-Jun activity in the bladder cancer tissues. We randomly selected 41 paraffin-embedded bladder tumor tissues from a total of 97 patient samples. The level of p-c-Jun was mostly in agreement on the pol ι expression (Fig. 4C). The expression of p-c-Jun was positively associated with elevated pol ι expression in bladder cancers (Table 3; r = 0.662, p<0.01). The result strengthens the conclusion that c-Jun positively regulates pol ι expression and suggests that abnormal p-c-Jun contribute to the dysregulated pol ι expression involved in the progression of bladder cancer.

**Table 3:** The expression of p-c-Jun was correlated with pol ι in bladder cancers.

Grade	L/L	I/I	Total No.
I	9	2	11
II	7	3	10
III	2	11	13
No.	18	16	34

There are 41 paraffin-embedded bladder tumor tissues from a total of 97 patient samples. The expression of pol ι coincided with p-c-Jun was 34 pairs of tissues. “L/L” was represented both low expression of p-c-Jun and pol ι; “I/I” was represented both high expression of p-c-Jun and pol ι. The number of samples was listed. The concordance of the p-c-Jun and pol ι expression was evaluated by bivariate test; p<0.01, r = 0.662.

### Overexpression of pol ι activated by JNK/c-Jun promotes UV-induced hypermutagenesis in T24 cells

To evaluate the significance of the overexpression of pol ι and p-c-Jun *in vivo*, we examined the potential hypermutagenic role in bladder cancer cells. As concerned that only the enzymatic properties of pol ι bypassing the UV lesion is relatively known, we are limit to distinguish if the potential hypermutagenesis outcome is mediated by pol ι through analyzing the mutation spectra of reporter genes damaged by UV. To gain the direct evidence that overexpressed pol ι mediated by elevated JNK activity is contributed to hypermutagenesis in cancer cells, we used the *SupF* gene-based shuttle vector (pSupFG1) DNA replication system *in vivo* [30], [31], [32]. We chose T24 cells line which is a malignant bladder cancer cells line and overexpresses pol ι protein to detect UV-induced mutations when the cells were pre-treated with or without SP600125. As shown in Table 4, there were a UV dose-dependent increase in the frequency of *SupF* gene mutants and an approximately three-fold increase in the mutation frequencies of T24 cells relative to those pretreated with the JNK inhibitor (p<0.01). For example, at the dose of 1000 J/m^2^, the mutation frequency was decreased from 9.8×10^−3^ to 3.3×10^−3^ in T24 cells pre-treated with SP600125. It is implied that elevated activity of JNK and overexpressed pol ι contribute to the hypermutagenesis in bladder cancer cells.

**Table 4:** Mutation frequency of *SupF* gene in T24 cells.

Cell line	Mutant No.	Total No.	Mutation Frequency (×10^−3^)*
1.Untreated plasmid			
T24	6	35100	0.171±0.056
T24+SP600125	6	36250	0.166±0.043
2. pSupFG1 plasmid treated with 500 J/m^2^			
T24	52	23126	2.249±0.448
T24+SP600125	33	27676	1.192±0.374
3. pSupFG1 plasmid treated with 1000 J/m^2^			
T24	249	25329	9.831±0.386
T24+SP600125	91	26981	3.373±0.343
4. pSupFG1 plasmid treated with 2000 J/m^2^			
T24	341	6650	51.278±1.886
T24+SP600125	143	7845	18.228±1.343

* Each data at least three independent experiments. The mutation frequency (×10^−3^) was described by mean± SE (SE: standard error).

**Figure 5:**
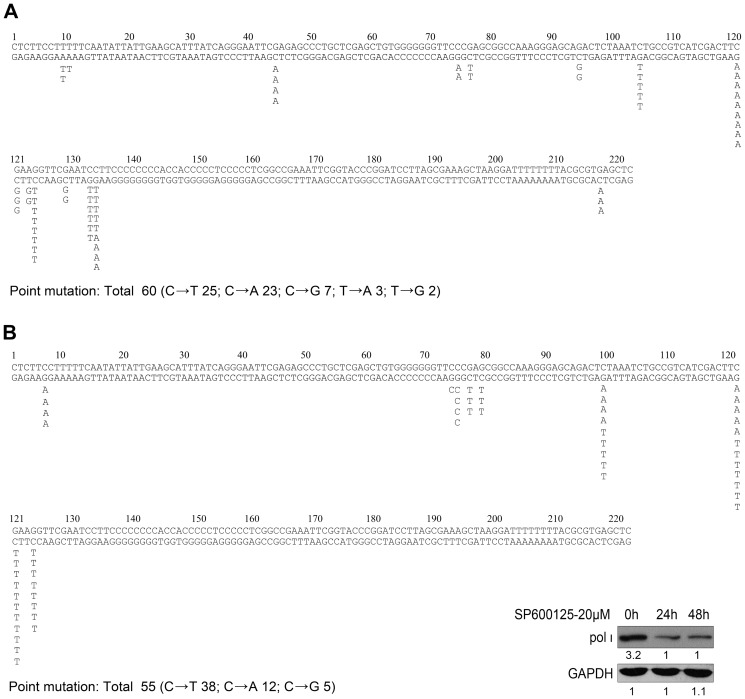
Mutation spectra generated by UVC (1000 J/m^2^) of *SupF* gene in T24 cells. (A) Mutations obtained from the T24 cells (total 60 point mutants in 25 bacterium samples). (B) Mutations obtained from the T24 cells pre-treated with SP600125 (total 55 point mutants in 25 bacterium samples). T24 cells treated by SP600125 two times each for 2 hours (one time every 24 hours for 48 hours) were lysed at 24 hours and 48 hours, and pol ι expression was analyzed by Western blot assay. Densitometric values were indicated below each panel. Point mutations were indicated under each base pair and the listed base represented a change from the top strand. *SupF* gene sequence was list in double strand.

To further address the hypermutagenesis is specially mediated by overexpressed pol ι, the mutation spectra was analyzed. There was a significantly different qualitative distribution of the type of transitions and transversions created around the UV-induced pyrimidine dimers between the T24 cells with or without JNK inhibitor treatment (Fig. 5). As the C→T transition being the predominant type (90%) of mutation in normal cells [33], there was a large increase in the number of transversion (Total transversion: 58.3%, including C→A, C→G, T→A and T→G) in T24 cells (Fig. 5A). This result of the mutation spectrum in T24 malignant cells was consistent with the enzymatic properties of pol ι from published *in vitro* kinetic analyses, which show pol ι prefers to misincorporate a G or T opposite the cyclobutane pyrimidine dimer and 6-4 pyrimidine pyrimidone photoadduct [6], [34]. This mutation spectrum indicates that pol ι certainly generates action in T24 cells. Following pre-treated with SP600125 two times each for 2 hours (one time every 24 hours for 48 hours) to ensure the maximum inhibition of JNK activity, not only was the expression of pol ι inhibited for 48 hours in T24 cells, the special mutation spectrum of pol ι was also notable decrease (Total transversion: 30.9%, including C→A and C→G) (Fig. 5B). Thus, it is suggested that overexpressed pol ι controlled by JNK/c-Jun plays an important role in mediating the lower fidelity and higher mutation frequency in bladder cancer cells.

## Discussion

In this study, we prove that activation of c-Jun directly enhances the basal and DNA damage induced expressions of pol ι in normal cells and bladder cancer cells. We further demonstrate that the overexpressed pol ι accompanied by elevated c-Jun phosphorylation was positively correlated with the clinical tumor grade in bladder cancer tissues. Our present study is the first report to directly determine the overexpression of pol ι in the investigation of human cancer tissues with statistical validation. Accordingly, the dysregulation of c-Jun has been reported in many different cancers, including its overexpression in bladder cancer [35], [36]. As the high heterogeneity and recurrence feature of bladder cancer [29], our current study supports the hypothesis that overexpression of pol ι induced by JNK/c-Jun plays a mutagenic role in urothelial cells carcinogenesis. Based on the current understanding of the enzymatic properties of pol ι, we are limited to only measure the UV-induced hypermutagenesis by the mutation spectra which is not obviously direct relevance for the development of clinical bladder cancer. There may be other damage agents or transcription factors to simultaneously regulate pol ι in complex mechanism *in vivo*. Nevertheless, our results still strongly suggest that dysregulated pol ι by c-Jun may be functioned as mutator, which provide the important clue to further clarify the hypermutagenic role of pol ι in response to other types of DNA damage agents in bladder cancers.

Despite extensive characterization of the error-prone feature of pol ι *in vitro* over the past decade, the cellular role of pol ι is still quite obscure from the limited *in vivo* published data. In response to different types of DNA damage, pol ι may carry out the TLS in different manners, as either error-free or error-prone. Studies involving *POLI* gene knockout in mice indicate that pol ι is not necessary for survival and can protect genome integrity [37], [38], [39]. Additionally, with the similar *SupF* reporter gene-based mutation detection system, pol ι has been shown to not be responsible for the mutagenesis resulting from UV damage in normal cells, such as HEK293 cells [40]. On the contrary, there are accumulated data suggesting that alterations of pol ι expression is associated with different cancer cells and can promote hypermutagenesis [5], [6], [7]. These seemingly conflicting data prompt us to raise the notion that only dysregulated or overexpressed pol ι can display a mutator phenotype. Together with the work of others, our results suggest that *POLI* gene may be a cancer susceptibility gene being special DNA damage induced mutation spectrum. Under normal circumstances, pol ι may be no effect or carry out error-free TLS to promote genomic stability. The pol ι-involved error-prone TLS of spontaneous mutation is highly regulated and limited, i.e., in the process of bypassing UV DNA lesions. However, dysregulated expression or increased activity of pol ι could result in an enhanced capacity for error-prone TLS. An increased capacity for error-prone TLS could provide cells with an advantage to cope with high-stress environments and lethal DNA damages, whereas the resultant hypermutagenesis may eventually lead to the development of malignant cancer cells.

With the potential hypermutagenesis role of overexpressed pol ι, it is important to explore its regulation mechanism. In this study, we firstly determine a novel transcriptional regulatory effect of MAPK/JNK/c-Jun signaling in pol ι-related error-prone TLS. Our results are consistent with plenty of published data explored the role of JNK/c-Jun pathways both in the DNA damage response and carcinogenesis. Moreover, this is also the first report to describe a functional link between the MAPK/JNK/c-Jun signaling cascades and the process of mutagenesis. It is suggested that over-activated JNK/c-Jun pathways may contribute to maintain genomic instability under DNA damage, which can eventually lead to carcinogenesis.

Up to date, although many studies examined the role of JNK pathway activation in DNA damage response is induced-apoptosis, the results are still contradictory. Persistent activation of JNK in response to cisplatin and UV treatment has been reported to be correlated with the induction of apoptosis. In contrast, a substantial number of reports support that JNK is anti-apoptotic [41]. Nevertheless, the majority of findings suggest that activated c-Jun provides a selective advantage by protecting human tumor cells from the cytotoxic effects of DNA damaging agents [42]. One manner which c-Jun could possibly influence cell survival to DNA damage response is by altering the cell capacity for DNA repair [43]. In support of this notion, there were many evidences that early cellular responses to DNA damage can strongly promote DNA repair capability by enhancing the transcription of different DNA repair genes [13], [44]. Our previous work has demonstrated that the induction of pol ι following UV irradiation is an early DNA damage response [4]. Based on our discovery of a novel transcriptional regulatory effect of MAPK/JNK/c-Jun signaling in pol ι-related error-prone TLS, our current study supports the notion that early activation of the MAPK/JNK/c-Jun signaling pathways plays a major anti-apoptotic role; in contrast, sustained signaling may confer pro-apoptotic effects, thereby promoting the occurrence of apoptosis [45].

A central question is whether JNK signaling is primarily stimulated by receptor-related or DNA damage-related mechanisms. It is well-documented that DNA damage can rapidly activate MAPK pathways by stimulating membrane growth factor and cytokine receptors, which likely occurs via a mechanism involving ROS formation and inhibition of tyrosine phosphatases [46]. However, mounting evidences suggest that DNA damage checkpoint or sustained arrest of DNA replication can induce the JNK pathway. It is reasonable to hypothesize that DNA damage can directly stimulate JNK pathway through nuclear signaling. In support of this notion, several reports have established that mutants exhibiting defective DNA repair differ from the wild-type cells with respect to JNK activation [26], [47], [48], [49]. ATR as a master regulator of DNA damage response [50] and XPC being an important DNA damage recognition protein regulating the cell cycle arrest and apoptosis [51], [52], [53] have been implicated in promoting early JNK activation during DNA damage response, but not sustained JNK activation [26], [49]. In this study, we provide the evidences that transient early JNK/c-Jun activation is largely responsible for pol ι induction and that DNA damage response protein ATR and XPC directly influence JNK pathway. To further address the roles of ATR and XPC in JNK signaling, our data imply that XPC and ATR can promote JNK activation via inhibiting MKP-1 expression, rather than activating the JNK kinases MKK4 and MKK7. Combined with others data [27], these findings prompt us to strongly suggest that early JNK/c-Jun activation serves a pro-survival function dependent on DNA damage response.

In summary, we propose that early JNK activation may directly promoted by activation of DNA damage response in an MKP-1-inhibition dependent manner. As a result of early JNK activation, pol ι mediating error-prone TLS process is regulated by the phosphorylation of c-Jun, whereas the over-activated JNK/c-Jun signaling or pol ι may contribute to genomic instability in bladder cancer cells. Our study offers a novel understanding of the role of pol ι or c-Jun as a mutator which may be potential target for anticancer treatment, and provides an insight into the relationship between TLS activation, JNK signaling and mutagenesis. In as much, the present study establishes an important basis for the future work, which will be addressed to answer the following important questions: (1) How to judge the role of pol ι or JNK pathway in hypermutagenesis mediated by others types of DNA damage which are more related to the carcinogenesis of bladder cancer? (2) What is the relationship between DNA damage induced JNK activation and traditional membrane associated signaling? (3) What is the fundamental mechanism for orchestrating the variety processes activated by JNK pathway in response to DNA damage?
